# Horizontal Gene Transfer in Plants and Implications for Biotechnology

**DOI:** 10.1002/pei3.70087

**Published:** 2025-09-21

**Authors:** Rojana Binte Azad, Shamfin Hossain Kasfy, Kutubuddin Molla, Tofazzal Islam

**Affiliations:** ^1^ Institute of Biotechnology and Genetic Engineering Gazipur Agricultural University Gazipur Bangladesh; ^2^ ICAR‐National Rice Research Institute Cuttack Odisha India

**Keywords:** bioengineering, biotechnological applications, climate‐smart crop development, eukaryotic evolution, eukaryotic horizontal gene transfer, HGT mechanism

## Abstract

Horizontal gene transfer (HGT), a fundamental process long acknowledged in prokaryotic evolution, is increasingly recognized as a pivotal force in shaping the evolutionary trajectories of eukaryotes, including plants. Despite its established significance in prokaryotic adaptation, the role of HGT in eukaryotic evolution is still understudied. HGT plays a pivotal role in the evolution of eukaryotes, giving rise to novel features that allow organisms to exploit new environments and resources with reduced competition. Moreover, the coevolution of interacting organisms in any ecosystem is greatly influenced by HGT. Recent discoveries of HGT events among eukaryotic species such as gene transfers from fungi to plants and from plants to whiteflies highlight the importance of understanding this phenomenon in the context of plant biology. In this review, we provide an update of recent findings related to plant and associated organisms like microorganisms, insects, and critically discuss the profound implications of HGT for plant evolution and adaptation, probing into potential underlying mechanisms, highlighting the knowledge gap and discussing their implications. In particular, we explore the potential applications of the new knowledge of HGT in plant biotechnology, illuminating its pivotal role in shaping the future landscape of bioengineering.

## 1 Introduction

Horizontal gene transfer (HGT), recognized as the lateral and nonsexual transmission of genetic material between organisms, has long been acknowledged as a fundamental mechanism in prokaryotic evolution. Initially documented in 1928 by British bacteriologist Frederick Griffith within prokaryotic organisms (Griffith 1928), HGT has since been extensively studied, revealing its pivotal role in microbial adaptation and evolution (Gogarten and Townsend 2005). Beyond prokaryotes, evidence suggests that HGT also plays a pivotal role in the evolution of eukaryotes, facilitating genetic adaptability and ecological diversification (Soucy et al. 2015; Van Etten and Bhattacharya 2020; Aubin et al. 2021). Unicellular eukaryotes have demonstrated remarkable adaptability through the acquisition of genes from diverse sources via HGT, expanding their ecological niches and thriving in various habitats (Keeling and Palmer 2008; Keeling 2009, 2024).

Once controversial, HGT in eukaryotes is now well‐supported by genomic evidence. Though far less frequent than in prokaryotes, HGT remains a significant evolutionary force in eukaryotes, shaped by methodological biases and constrained by complex cellular barriers (Keeling and Palmer 2008). Despite these challenges, the endosymbiotic theory exemplifies the intricate relationship between prokaryotes and eukaryotes, underscoring the potential for genetic exchange between these domains of life. Plastids, the hallmark of photosynthetic eukaryotes, are derived from cyanobacterial endosymbionts in a eukaryotic host (Keeling 2024). The establishment of cyanobacterial endosymbionts or plastids triggered the origin of Plantae, such as red algae, glaucophytes, and green plants (Van Etten et al. 2024). The origin of the major phytohormone auxin synthesis and vascular development in plants is thought to be associated with HGT (Yang et al. 2015). Recent studies reveal that successful HGT events in plants have driven adaptation and diversification, enabling the evolution of novel traits that open new ecological niches and trigger adaptive radiations with reduced competition (Soucy et al. 2015; Ma et al. 2022; Van Etten et al. 2024).

Multiple lines of evidence suggest that HGT spans across various domains of life, influencing ecosystems and the fitness of recipient organisms (Savory et al. 2015; Van Etten and Bhattacharya 2020; Aubin et al. 2021). From bacteria to plants, plants to insects, fungi to plants, and even from plants to parasitic plants, HGT exerts significant impacts on plant biology (Schell et al. 1979; Yoshida et al. 2010; Wang et al. 2020; Xia et al. 2021). Recent discoveries, such as the transfer of the *Fhb7* gene from a fungus to a wild relative of wheat (Wang et al. 2020), and the acquisition of phenolic glycoside detoxification enzymes by whiteflies from plants (Xia et al. 2021), underscore the widespread occurrence of HGT in eukaryotes, especially plants. Notably, the acquisition of RNA‐guided endonucleases like Fanzor by diverse eukaryotic organisms, including plants from prokaryotes, suggests a complex interplay mediated by viruses or eukaryotic symbionts (Saito et al. 2023). This intricate exchange highlights the dynamic nature of genetic flux and its profound implications for the evolution of diverse life forms, particularly plants. In spite of these discoveries, the precise mechanisms governing HGT in plants and other eukaryotic organisms remain largely unknown. However, uncovering these mechanisms presents an exciting opportunity for advancing plant biotechnology and bioengineering, with the potential to transform our capacity to manipulate genetic material for beneficial purposes (Islam et al. 2023). The natural gene‐transfer mechanism of *Agrobacterium tumefaciens* has long been instrumental in plant genetic engineering, facilitating the transfer of desired genes for crop improvement that significantly impacts global food security (Costantino et al. 1980; Rahman et al. 2024).

HGT in plants has largely been studied from an evolutionary perspective (Van Etten and Bhattacharya 2020; Aubin et al. 2021; Pereira et al. 2022; Keeling 2024), yet a comprehensive review of its biotechnological potential is still lacking. This review aims to address this gap by providing updated insights into the prevalence, functional impact, and existing knowledge of HGT in eukaryotes, with an emphasis on plants. Additionally, it explores the biological implications of these events and their potential applications in biotechnological innovation, offering avenues for harnessing HGT for advancements in bioengineering and plant biotechnology for climate‐smart agriculture. By elucidating the significance of HGT in eukaryotes, this review seeks to stimulate further research and practical applications in diverse biological contexts of plant communications.

### 1.1 Prevalence and Functional Dynamics of HGT in Plant

#### 1.1.1 HGT Between Plant and Virus

Horizontal Gene Transfer from viruses to plants is a remarkably prevalent mechanism, evidenced by the widespread integration of endogenous viral elements (EVEs) into host genomes. The most common of these are endogenous caulimovirids (ECVs), ancient viral DNA now found scattered across the genomes of numerous vascular plant species (Vassilieff et al. 2023). While this gene flow is less frequent in plants than in unicellular organisms, likely due to the plant germline barrier, the sheer number of transfers is still significant (Irwin et al. 2022).

These integrated genes are not merely genomic fossils; they are major drivers of plant evolution. A striking example is the acquisition of a viral cellulose synthase gene, which is correlated with the evolution of the cellulose‐based cell wall in Streptophytes, the ancestors of all land plants (Irwin et al. 2022). This supports the finding that plant genomes contain genetic “footprints” from past infections by giant viruses, demonstrating that HGT from viruses has been fundamental in shaping core plant biology (Moniruzzaman et al. 2020). The abundance of these integrated viral genes, including sequences identified in banana, rice, and Arabidopsis, represents a significant natural reservoir of genetic material that plants co‐opt for novel functions, offering a promising frontier for biotechnological discovery (Aubin et al. 2021; Chen et al. 2024).

#### 1.1.2 HGT Between Plant and Bacteria

**FIGURE 1:**
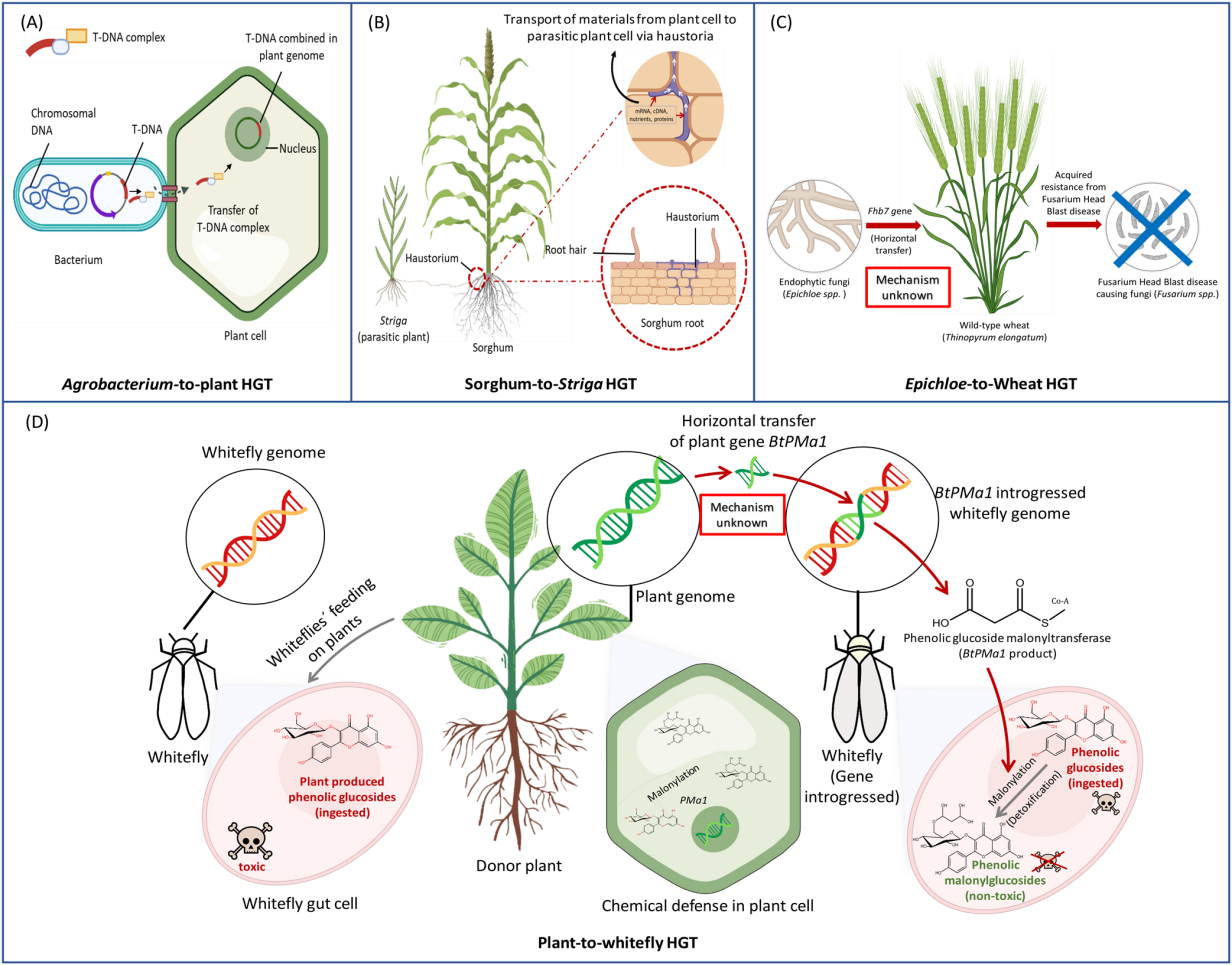
Notable HGT events in eukaryotes (A) *Agrobacterium*, a naturally occurring genetic engineer, can transfer its T‐DNA from a Ti plasmid to a target plant cell by using accessory components to build a T‐DNA complex. This transfer is direct, as the complex is delivered straight from the bacterium to the plant cell. (B) *Striga* establishes a connection with the Sorghum (host) root system through haustoria, which enable the transfer of nutrients, water, and genomic components like mRNA and cDNA from the host to the parasite. (C) The horizontal transfer of the *Fhb7* gene from an endophytic fungus, *Epicloe*, to wild‐type wheat *Thinopyrum elongatum* has been demonstrated to be beneficial in combating Fusarium Head Blight disease (FHB). However, the precise mechanism of this HGT is not yet fully understood. (D) Whitefly has infiltrated the plant defense system after acquiring some of these genes (*BtPMaT1* and other genes) from plants via HGT, the mechanism of which still remains unknown.

Horizontal Gene Transfer from bacteria has been a major force in plant evolution, providing novel genes that have shaped plant genomes and driven key adaptations over millions of years (Cheng et al. 2019; Van Etten and Bhattacharya 2020; Aubin et al. 2021). The genome of the fern *Ceratopteris richardii* , for example, shows significant changes from bacterial gene transfers over the past 60 million years (Marchant et al. 2022). These transfers, often from symbiotic soil bacteria, have been critical for evolutionary milestones; for instance, the acquisition of stress tolerance genes from bacteria is linked to the successful transition of plants to land (Cheng et al. 2019). HGT also provides direct advantages, such as an insect resistance gene found in ferns like *Azolla* that was likely acquired from bacteria (Li, Brouwer, et al. 2018; Li, Zhao, et al. 2018). A remarkable and well‐studied example of HGT is the interaction between the soil bacterium *Agrobacterium tumefaciens* and plants. *Agrobacterium* causes crown gall disease (plant tumors) by performing an interkingdom genetic transfer (Smith and Townsend 1907; Costantino et al. 1980). Pathogenic strains carry a tumor‐inducing (Ti) plasmid, from which a specific segment called T‐DNA is transferred and integrated directly into the plant's genome (Figure 1A) (Schell et al. 1979; Gelvin 2003; Pereira and Dunning 2023). This T‐DNA essentially hijacks the plant's cellular machinery, forcing it to produce tumors and unique compounds called opines. These opines, which are amino acid derivatives, serve as an exclusive carbon and nitrogen source for the bacteria, a process aptly termed “genetic colonization” (Schell et al. 1979). The cultivated sweet potato stands as an intriguing example of an *Agrobacterium*‐mediated natural transgenic food crop, with its genome accommodating T‐DNAs carrying expressed genes (Kyndt et al. 2015). This natural mechanism is so efficient that it has become the foundation for modern plant biotechnology.

However, recent studies reveal a more complex dynamic. Contrary to earlier assumptions, the rate of HGT does not necessarily increase when bacteria share a niche with a plant, and the gene flow is highly asymmetric, with significantly more genes moving from bacteria to plants than the reverse. These transferred genes often provide functional advantages, such as the ability for bacteria to colonize plants by breaking down cell wall sugars (Haimlich et al. 2024). This intricate exchange has fundamentally shaped the interconnected metabolisms of both kingdoms and continues to provide new perspectives on their co‐evolution.

#### 1.1.3 Horizontal Gene Transfer Between Plant and Fungi

Horizontal Gene Transfer between plants and fungi is a dynamic, bidirectional exchange of genetic material that significantly shapes their evolution, creating novel adaptations for both symbiosis and conflict. In one direction, plants have acquired novel defensive capabilities by incorporating fungal genes, ranging from ancient mitochondrial DNA (Vaughn et al. 1995), to the first documented case of horizontal transfer of a nuclear gene from a distantly related eukaryote into flowering plants, revealing a novel adaptive strategy in angiosperms (Shinozuka et al. 2017), and more recently to glycosyl hydrolase (GH) genes that expand and diversify enzyme functions (Kfoury et al. 2024). A prime example involves a case of lateral gene transfer between a fungus and a species of wheat. The wild relative of wheat, *Thinopyrum elongatum* , has acquired a fungal gene, *Fhb7*, from the endophytic fungus *Epichloe*. This gene equips the wheat plant with defenses against *Fusarium* sp., the causative agent of a destructive Fusarium head blight (FHB) disease in wheat (Wang et al. 2020). The *Fhb7* gene encodes a glutathione‐*S*‐transferase (GST) enzyme capable of detoxifying trichothecene toxins produced by *Fusarium* sp. This discovery not only enhances our understanding of resistance gene evolution but also suggests a novel approach to identifying resistance genes. The discovery of this gene has been opened an opportunity for breeding FHB resistant wheat (Figure 1C).

Conversely, HGT also fuels the adaptation and host‐specific pathogenicity of fungi. For instance, pathogenic *Colletotrichum* fungi became more effective at infecting plants after acquiring a plant‐like subtilisin gene (Armijos Jaramillo et al. 2013), while the symbiotic fungus *Rhizophagus irregularis* integrated functional genes from plants to enhance its symbiotic lifestyle (Li, Brouwer, et al. 2018; Li, Zhao, et al. 2018). The impact of HGT extends even further, as it has also facilitated the evolution of plant parasitic mechanisms in fungus‐like oomycetes (Savory et al. 2018). HGT plays a pivotal role in shaping the host‐specific pathogenic traits and adaptability of fungal species. Blast disease caused by several pathotypes of *Pyricularia oryzae* (synonym: *Magnaporthe oryzae*) is the most destructive pathogen of major food crops such as rice, wheat, maize, and millets and thus is considered one of the major threats to global food security (Islam et al. 2016, 2020). The pathogenic strain of *P. oryzae* pathotype *Triticum* causing fearsome wheat blast has acquired genes from related species through HGT, contributing to the establishment of a fungal niche (Kobayashi et al. 2023). Scientists suggest that epigenetic modifications are crucial for domesticating these transferred genes, though the precise mechanisms driving HGT in plant pathogens still warrant further investigation.

#### 1.1.4 HGT in Plantae

Horizontal Gene Transfer between plants is most frequently documented in parasitic species like *Cuscuta*, *Rafflesia*, and *Striga* (Davis and Xi 2015; Aubin et al. 2021), where haustorial feeding linkages between parasites and their hosts facilitate close cellular interactions, promoting macromolecule exchanges that result in stable genetic component exchanges through HGT (Figure 1B). A classic example is *Striga hermonthica* (a parasitic weed), which acquired the nuclear gene ShContig948 from its host, *Sorghum bicolor* . The presence of a poly‐A‐like sequence at the gene's 3′ end strongly suggests the transfer occurred via an mRNA or cDNA intermediate. This HGT event is thought to be relatively recent, occurring soon after the speciation of the Striga genus (Yoshida et al. 2010). Research indicates that HGT happens more frequently in parasitic species than in free‐living plants and is most common in mitochondrial genomes compared to plastid or nuclear genomes (Yoshida et al. 2010, 2016).

However, HGT is not confined to parasitism and has been proven by systematic genomic scanning to be a widespread phenomenon in grasses, affecting a majority of species studied, including major crops like maize and wheat (Wickell and Li 2020; Hibdige et al. 2021). A striking example is the grass *Alloteropsis semialata*, which acquired 59 functional genes for photosynthesis, disease resistance, and stress tolerance from at least nine different donor grasses. Recent research has identified key factors that facilitate such transfers: species with rhizomes acquire significantly more genes, likely because this growth habit boosts opportunities for transfer into the germline, and the frequency of successful transfers decreases with phylogenetic distance, suggesting genomic compatibility is crucial. In addition to these pathways, natural grafting has also been identified as a mechanism for HGT that can even lead to the formation of new allopolyploid species (Hibdige et al. 2021). Ultimately, the horizontal acquisition of genes has multifaceted impacts on terrestrial plants, influencing their stress responses and metabolism, with the success of this powerful evolutionary force depending on both the opportunity for transfer and compatibility between species (Ma et al. 2022).

#### 1.1.5 HGT Between Plant and Insect

Similar to microorganisms and plants, many plant‐feeding insects and mites possess genes resulting from HGT events (Table 1) (Pauchet et al. 2014; Xia et al. 2021). The discoveries of RIP‐producing genes transfer from plants to whiteflies by Lapadula and colleagues has opened a new dimension to eukaryotic HGT (Lapadula et al. 2017, 2020). Whiteflies are polyphagous pests of more than 600 species of plants. An extraordinary instance of HGT from plant to insect is the lateral transfer of two plant toxin detoxifying genes within the genomes of two cryptic insect species of the sweet potato whitefly ( *Bemisia tabaci* ) (Figure 1D). One of these genes, *BtPMaT1*, is known to encode a phenolic glycoside malonyltransferase, which enables the whitefly to detoxify the plant's chemical defense compounds phenolic glycosides during feeding on plants (Xia et al. 2021). Before this discovery, it remained unclear how insects with diverse host ranges defended against toxic phenolic glycosides. However, HGT of the detoxifying gene explains only a part of the story of the polyphagous nature of whiteflies, indicating the presence of additional unknown mechanisms and genes at play. Furthermore, a comprehensive genomic analysis of whiteflies revealed at least 55 genes across three cryptic species of *B. tabaci* that share greater similarity with plant genes in terms of their amino acid composition (Gilbert and Maumus 2023). Most of these newly discovered genes are believed to be associated with plant‐insect interactions based on their predicted functions. This phenomenon, where polyphagous insect pests acquire plant genes, highlights the intricate relationships between plants and their insect pests, underscoring the potential of HGT as a survival strategy for these pests. Understanding the underlying molecular mechanisms involved in this process can pave the way for the development of novel biotechnological approaches to manage polyphagous insect pests sustainably, ensuring crop protection while minimizing environmental pollution due to the application of insecticides (Islam et al. 2023).

**TABLE 1:** Some landmark examples of HGT in plants and associated insects and microbes showcasing its functional significance in diverse biological contexts.

Donor organism	Gene transferred	Recipient organism	Functional significance of the HGT event	References
*Agrobacterium* spp. (Bacteria)	T‐ DNA	Plant	T‐DNA induce the exchange of signal and compound secretion in altered plants; A developed tool in biotechnology	Schell et al. (1979), Gelvin (2003)
Fungus (*Epichloe*)	*Fhb7* Gene	Wild relative of wheat ( *Thinopyrum elongatum* )	Gene synthesizes enzyme to detoxify the fungal toxin; Source for resistance breeding	Wang et al. (2020)
Plant	*BtPMaT1and BtPMaT2*	Whitefly	*BtPMaT1* Neutralizes a plant toxic (phenolic glycosides) and function *BtPMaT2* of is unknown; Involve in host‐insect interaction	Xia et al. (2021)
Plant	Multiple genes related to plant‐insect interaction	Insects like whitefly, aphid and lepidopterans	HT genes have roles in insect life, spanning immunity, adaptability, and even lepidopteran courtship behavior.	Gilbert and Maumus (2023); Li et al. (2022)
Plant	RIP‐ gene	Whitefly	Ribosome‐inactivating proteins (RIPs) in whiteflies are thought to play a role in the insect's defense against pathogens.	Lapadula et al. (2017, 2020)
Sorghum	*ShContig9483*	*Striga hermonthica*	Gene encodes a 448 amino acid protein with unknown function; Clear evidence of nuclear HGT	Yoshida et al. (2010)
Fungus	ß‐1,6‐glucanase gene	Perennial ryegrass	Synthesized enzyme helps in fungal cell wall degradation; Factor of plant response to pathogen; First instance of HGT of Nuclear gene between two taxonomically distant eukaryotes	Shinozuka et al. (2017)
Fungus	GH‐ gene	Plant	Encoded enzymes are related to plant cell wall polysaccharide metabolism; Factor for host–microbe interaction	Kfoury et al. (2024)
Plant	Subtilisin gene	Fungus (*Colletotrichum*)	Contribute in plant infection	Armijos Jaramillo et al. (2013)
Bacteria	Fz (Fanzor protein)	Eukaryotes (Plant, fungus, human)	Eukaryotic programmable RNA‐guided endonucleases; a new tool for genome editing	Saito et al. (2023)

HGT between plants and insects is not limited to whiteflies. A recent comprehensive analysis of 218 high‐quality insect genomes has shown that insects have acquired 1410 genes from 670 unique donors, including 25 plant species (Li et al. 2022). While lepidopterans demonstrate the highest rate of horizontally acquired genes, hemipteran insects, such as whiteflies and aphids, closely follow suit. Functional studies on these 1410 genes have revealed their significant roles in insect life, spanning immunity, adaptability, and even lepidopteran courtship behavior. HGTs are typically thought to retain their ancestral functions, but a recent study by Hu et al. (2025) challenges this view by exploring a plant‐to‐insect gene transfer event where a thaumatin‐like protein (TLP) having antifungal activity transferred from plants to two whitefly species *Trialeurodes vaporariorum* and *Bemisia tabaci* . In *T. vaporariorum* , TLP functions similarly to its plant origin, playing an antifungal role. However, in *B. tabaci* , TLP has evolved into an effector that suppresses plant immune responses. This divergence suggests that horizontally acquired genes can evolve distinct, species‐specific functions over time. These findings offer a new perspective on the role of HGT in driving unique evolutionary trajectories.

## 2 Mechanisms of HGT

**FIGURE 2:**
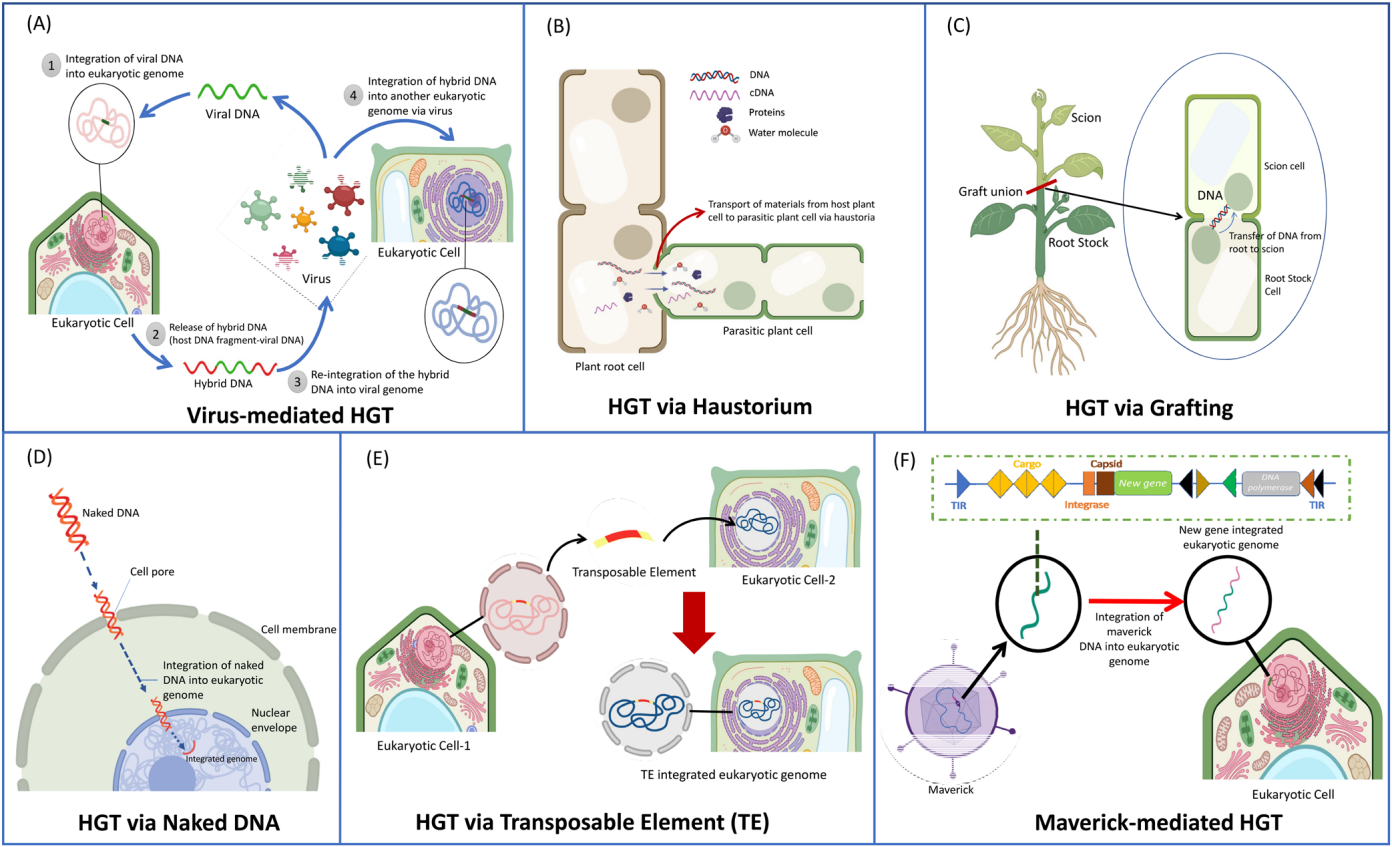
Known and putative mechanisms of HGT between plants and other organisms. (A) Horizontal transfer, facilitated by viruses, can be conceptualized as a two‐stage process: Initially, the virus obtains a fragment of the host genome, followed by the integration of this fragment into the genome of another host species. (B) The parasitic plant forms a connection with the host root system via haustoria, facilitating the exchange of nutrients and genomic elements such as mRNA and cDNA from the host cell to the parasite cell. (C) The mechanism of HGT via grafting involves the translocation of genomic materials (DNA) from the root stock cell to the scion cell through the grafting union, facilitating the exchange of genetic information. (D) As a mechanism of horizontal gene transfer, cell poration facilitates the entry of naked DNA from the environment into another cell's chromosome, enabling the integration of foreign genetic material. (E) Transposable Elements (TE) are DNA fragments that have the inherent ability to move and integrate back into chromosomes. (F) Mavericks is characterized by a blend of traits from both transposons and viruses. It features terminal inverted repeats at their extremities and demonstrates the capacity to transpose and integrate within genomes.

Although occurrences of HGT between plants and various prokaryotic and eukaryotic organisms are now well founded, however, mechanisms of this HGT remain vastly unknown. Recent progress in methodologies for omics and computational biological investigation seems to open these black boxes that are crucial for understanding HGT's transformative role in shaping genetic diversity and evolutionary trajectories. Several previous hypotheses suggest that HGT in plants occurs through diverse pathways (Pereira et al. 2022; Pereira and Dunning 2023), including viral integration, host–parasite interactions (Mishina et al. 2023), grafting, environmental DNA uptake, and mobile genetic elements like transposons, mavericks. These processes involve intricate steps where foreign DNA penetrates cells, crosses the nuclear envelope, and integrates into the genome. Direct cellular contact or facilitation by intermediaries may enable these transfers. The known and putative mechanisms of HGT are summarized and illustrated in Figure 2.

*Virus‐mediated gene transfer* occurs through endogenization (Figure 2A), where viral DNA or reverse‐transcribed RNA is incorporated into host germline genomes, forming EVEs. Retroviruses, uniquely equipped with enzymatic machinery for autonomous integration, are the primary contributors to EVEs, which are vertically inherited and fixed within populations (Gilbert and Cordaux 2017; Irwin et al. 2022). RNA viruses are the predominant source of EVEs, and their integration has been widely documented across plants, arthropods, and algae. In plants, families like *Geminiviridae* and *Potyviridae* have been implicated. Endogenous non‐retroviral elements (ENREs), derived from double‐stranded RNA viruses, are rarer but still notable contributors, with *Partitiviridae* and *Chrysoviridae* frequently identified (Catoni et al. 2018). Integrated viral genes may confer novel functions, influencing eukaryotic evolution by introducing adaptive traits. Additionally, bacteria like *Agrobacterium* transfer genetic material into host plants via “*Agrobacterium*‐mediated transformation,” a process discussed earlier (Figure 1A).

*Host–parasite connections* offer another HGT route, particularly through the haustoria of parasitic plants. These specialized structures enable parasitic plants to invade host tissues, forming a physical and biochemical bridge (Figure 2B). Through this connection, water, nutrients, proteins, nucleotides, and genetic material, such as mRNA and retrotransposons, can be exchanged. Such genetic exchanges have profound implications for the co‐evolution of host–parasite systems, driving genomic innovation and adaptation (Yoshida et al. 2016). A recent study of massive HGTs from a host insect to a parasite hairworm that manipulates host behavior may help to understand the adaptive significances of HGTs occurring in host–parasite systems (Mishina et al. 2023).

*Grafting*, a widely used agricultural practice, facilitates genetic material exchange between stock and scion (Figure 2C). Experiments in *Nicotiana* species have shown that grafting can result in the transfer of nuclear, mitochondrial, and plastid genomes, with chloroplast DNA being exchanged through cell wall pores. This phenomenon, known as “plastid capture,” highlights grafting as a potential natural mechanism for HGT (Fuentes et al. 2014; Hibdige et al. 2021).

*Naked environmental DNA* provides another reservoir for HGT. DNA adsorbed onto minerals can persist for extended periods, forming stable reservoirs available for integration into other organisms (Figure 2D). Studies suggest that this process, supported by DNA‐mineral interactions, may facilitate genetic exchanges across temporal and spatial scales, thereby contributing to evolutionary innovation (Sand and Jelavić 2018).

*Transposons*, particularly transposable elements (TEs), play a central role in HGT across plants, fungi, oomycetes, and animals (Savory et al. 2015; Pulido and Casacuberta 2023). TEs such as LTR‐retrotransposons, DNA transposons, and non‐LTR‐retrotransposons are mobile genetic elements capable of transferring genes across species (Figure 2E). Over 3000 cases of horizontal transposon transfer (HTT) have been reported, with TEs often associated with tissue damage, insect‐mediated transfer, and root cell interactions (Dotto et al. 2015). These elements contribute to chromosomal rearrangements, genetic diversity, and novel functions, as exemplified by TE exchanges between rice and millet (El Baidouri et al. 2014).

*Mavericks*, a unique class of transposable elements, exhibit dual characteristics of transposons and viruses. These large elements carry genes for structural and enzymatic functions, enabling their integration into genomes (Figure 2E). In nematodes, Mavericks have acquired fusogen proteins that enhance their ability to mediate HGT across reproductive barriers (Widen et al. 2023). Similarly, “starships,” recently discovered in fungi, are mobile genetic elements that carry large gene cargoes and promote lateral gene transfer (Gluck‐Thaler et al. 2022). While their presence in plants remains unverified, they represent a promising avenue for future research on HGT mechanisms.

## 3 Biotechnological Implications of HGT Knowledge

### 3.1 Bioengineering

**FIGURE 3:**
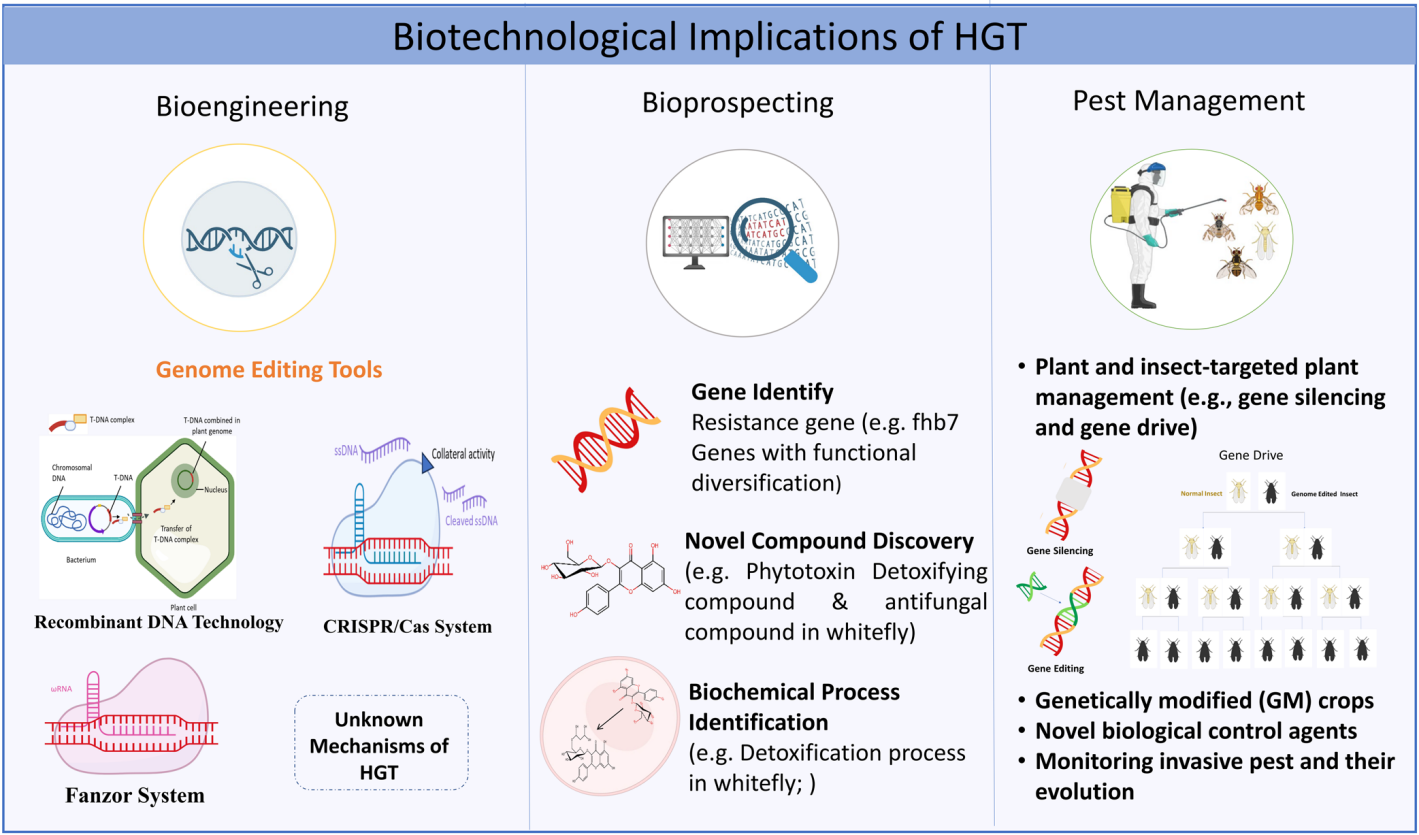
Potential biotechnological applications of the insights into eukaryotic HGT.

Understanding the mechanisms and prevalence of HGT in eukaryotes carries significant implications for plant biotechnology. By comprehending the intricacies of HGT, researchers can devise more effective gene transfer techniques to introduce desired traits into eukaryotic organisms. Three prominent examples of HGT applied in bioengineering are: (i) Recombinant DNA technology (ii) CRISPR‐Cas system and (iii) Fanzor‐mediated genome editing (Figure 3). *A. tumefaciens* has long served as a valuable tool in bioengineering due to its unique ability to transfer genetic material into plant cells. Leveraging this natural mechanism, researchers have successfully introduced desired genes into plants, leading to the development of modern crop improvement tools. Over the past few decades, this approach has led to substantial improvements in the yield of crops such as corn, soybean, cotton, and numerous other economically significant food and cash crops worldwide (Rahman et al. 2024).

CRISPR‐Cas provides another notable illustration of HGT, incorporating fragments of viral genomes into bacterial DNA (Jinek et al. 2012), thus playing a crucial role in bacterial immunity. This natural process has been ingeniously harnessed to develop CRISPR technology, enabling precise genome editing and molecular diagnostics with numerous applications in agriculture and medicine (Molla et al. 2020; Wang and Doudna 2023). Furthermore, Fanzor, an RNA‐guided endonuclease, is presumed to result from horizontal gene transfer, originating from bacterial genomes and later integrating into eukaryotic genomes. Its precise DNA editing capability renders it as a promising tool for various applications in biotechnology and medical research (Saito et al. 2023).

The elucidation of mechanisms in Recombinant DNA technology, CRISPR‐Cas, and Fanzor has facilitated their effective utilization as bioengineering tools, significantly contributing to synthetic biology research and development. By unraveling the fundamental principles governing HGT processes in plants and other eukaryotes, researchers may unlock new avenues for innovative tools and techniques. Such insights could empower synthetic biologists to design and engineer organisms. Additionally, understanding HGT mechanisms can inform the development of gene therapy approaches, enabling more efficient delivery and integration of therapeutic genes. Therefore, a deeper understanding of HGT mechanisms holds immense potential for the future of bioengineering worldwide for ensuring food and nutritional security under the changing climate.

### 3.2 Bioprospecting

Understanding HGT in eukaryotes is instrumental in bioprospecting, the systematic exploration of biological resources like plants, animals, and microorganisms to uncover novel compounds, genes, and biochemical processes with prospects (resistance, therapeutic, and commercial potential) (Figure 3). By delving into the genetic diversity resulting from HGT events, researchers can pinpoint valuable genetic reservoirs, fostering innovation in biotechnology. For instance, identifying the HGT event involving the *Fhb7* gene from fungi to plants has not only elucidated resistance gene evolution mechanisms but has also enabled the development of FHB resistant wheat varieties with introgressed *Fhb7* genes (Wang et al. 2020). This discovery sheds light on resistance mechanisms in plants and introduces a novel approach for identifying resistance genes. Furthermore, understanding how genes are transferred between eukaryotic organisms enables the identification of unique genetic pathways and the bioactive compounds they produce, potentially leading to drug discoveries and other valuable applications. HGT can also result in the production of known bioactive compounds in previously unexplored organisms. Here, the discovery of a plant toxin‐detoxifying compound and its associated pathway in whiteflies as a result of HGT from plants (Xia et al. 2021) is considered a striking example of it. Additionally, studying HGT between eukaryotes offers insights into evolutionary relationships and genetic exchanges among different organisms. This information guides bioprospecting efforts towards specific taxa or ecosystems with a higher likelihood of yielding novel compounds or genes of interest. Notably, evidence of functional diversification in horizontally transferred genes such as those observed between whiteflies and plants (Hu et al. 2025) highlights the potential for discovering genes with unique or enhanced functions in recipient organisms. These findings open up promising opportunities for identifying genes of interest with specialized or differential roles. Researchers can leverage genes acquired through HGT that encode therapeutic proteins or enzymes, thus enhancing pharmaceutical production in eukaryotic systems. Moreover, HGT rapidly alters bacterial genetics, enabling the evolution of new catabolic abilities, and the spread of these genes among microbial populations offers an alternative to bioaugmentation in bioremediation (Bhandari and Karn 2019). Similarly, eukaryotic HGT holds equal significance in environmental biotechnology, contributing to the development of bioremediation strategies for effective environmental cleanup.

### 3.3 Pest Management

Identifying the mechanisms of HGT through which crops and pests acquire new genetic material laterally can pave the way for targeted interventions using gene silencing techniques or gene editing tools like CRISPR‐Cas (Figure 3) (Islam et al. 2023). This knowledge can also aid in predicting the emergence and spread of new crop‐harming pests, enabling proactive measures to prevent infestations. Moreover, understanding the role of HGT in pests' acquisition of resistance to plant toxins can facilitate the development of genetically modified crops with enhanced resistance to these pests. This may involve bioengineering plants to produce higher levels of insect inhibitory metabolites or employing gene editing techniques to modify the genes responsible for detoxifying plant toxins in pests. In recent years, significant progress has been made in developing CRISPR‐based gene drives to promote the biased inheritance of specific traits, offering a powerful tool to disrupt pathogen transmission or control insect populations. An insect‐targeted strategy has been proposed for controlling whitefly, involving gene drive technique to inactivate or silence genes involved in detoxifying plant toxins (Islam et al. 2023). As a result, the edited insects in subsequent generations would lose the ability to feed on toxin‐producing plants, leading to a reduction in pest populations and crop damage. Such approaches can contribute to more sustainable and ecologically sound pest management strategies for eco‐friendly agriculture and food security.

## 4 Ethical Considerations and Risk Assessment of HGT‐Derived GM Crops

The growing understanding of HGT in eukaryotes, particularly between plants and insects, while promising for plant biotechnology, also introduces significant ethical considerations and potential risks for genetically modified (GM) crop development (Philips et al. 2022; Noack et al. 2024). A primary concern is the unpredictable nature of gene integration and expression when employing less understood HGT mechanisms, which could lead to unforeseen metabolic alterations, growth anomalies, or the creation of novel allergens and toxins in GM plants. Furthermore, the pervasive nature of HGT within ecosystems raises the possibility of unintended lateral transfer of engineered genes to non‐target organisms, such as wild crop relatives or beneficial insects, potentially resulting in “superweeds” or disrupted ecological balances (Courtier‐Orgogozo et al. 2020). The observed functional diversification of HGTs further complicates risk assessment, as an engineered gene's function in a new host might deviate significantly from its original intent. Therefore, robust and comprehensive risk assessment frameworks, encompassing meticulous environmental impact studies and diligent post‐market monitoring, are indispensable to ensure the responsible, ethical, and sustainable application of HGT‐derived knowledge in the advancement of GM crop technology (Philips et al. 2022; Noack et al. 2024).

## 5 Concluding Remark and Prospects

HGT events between plants and other organisms carry significant functional implications, especially during major ecological transitions. It is imperative to shift our focus from whether HGT affected eukaryotes to understanding how it influenced their ecology and evolution. An illustrative example of this concept is evident in the coevolutionary arms race between plants and whiteflies, highlighting the occurrence of HGT within this dynamic relationship. Delving into how genetic material is transferred between different eukaryotic species and integrated into recipient genomes can yield valuable insights into the processes driving genetic diversity and unlock new avenues for bioengineering. Nevertheless, most instances of HGT observed so far have been linked to conserved functionality. However, very recent evidence on the potential for functional diversification of the transferred genes opens a new perspective in HGT research. Understanding how these horizontally transferred genes diversify across species could lead to groundbreaking discoveries, unveiling their multifaceted roles and applications. Furthermore, comprehensive studies that compare HGT across diverse eukaryotic lineages using consistent parameters are necessary. Standardizing parameters and methodologies for studying HGT in eukaryotes is critical for ensuring comparability and reproducibility across studies. Until now, there is no reliable method of determining the origin and time period of the HGT events. Establishing consistent criteria for identifying and analyzing HGT events can aid in drawing meaningful conclusions about the prevalence and impact of genetic transfer in eukaryotic genomes. With the growing wealth of genomic data, advanced bioinformatics tools are crucial for managing biological data effectively. Additionally, the anticipated implications of HGT in eukaryotes necessitate experimental validation to assess their effectiveness. This holds promise for the development of climate‐smart biotechnologies aimed at sustainable management in agriculture, the environment, and beyond.

## Conflicts of Interest

The authors declare no conflicts of interest.

## Data Availability

The authors have nothing to report.
